# How can care settings for people with intellectual disabilities embed health promotion?

**DOI:** 10.1111/jar.12776

**Published:** 2020-07-06

**Authors:** Kristel Vlot‐van Anrooij, Monique C. J. Koks‐Leensen, Anneke van der Cruijsen, Henk Jansen, Koos van der Velden, Geraline Leusink, Thessa I. M. Hilgenkamp, Jenneken Naaldenberg

**Affiliations:** ^1^ Department of Primary and Community Care, Intellectual Disabilities and Health Radboud University Medical Center, Radboud Institute for Health Sciences Nijmegen The Netherlands; ^2^ Department of Primary and Community Care Radboud University Medical Center, Radboud Institute for Health Sciences Nijmegen The Netherlands; ^3^ Department of General Practice, Intellectual Disability Medicine Erasmus MC, University Medical Center Rotterdam The Netherlands; ^4^ Department of Physical Therapy University of Nevada Las Vegas NV USA

**Keywords:** health assets, health promotion, inclusive research, intellectual disability, settings approach

## Abstract

**Background:**

People with intellectual disabilities (ID) depend on their environment to live healthily. Asset‐based health promotion enhances a settings’ health‐promoting capacity starting with identifying protective or promotive factors that sustain health.

**Method:**

This inclusive mixed‐methods study used group sessions to generate and rank ideas on assets supporting healthy nutrition and physical activity in Dutch intellectual disability care settings. Participants included people with moderate intellectual disabilities and family and care professionals of people with severe/profound intellectual disabilities.

**Results:**

Fifty‐one participants identified 185 assets in group sessions. They include the following: (i) the social network and ways “people” can support, (ii) assets in/around “places,” and person–environment fit, and (iii) “preconditions”: health care, prevention, budget, and policy.

**Conclusion:**

This inclusive research provides a user perspective on assets in the living environment supporting healthy living. This gives insight in contextual factors needed for development and sustainable embedment of health promotion in the systems of intellectual disability support settings.

## 1 INTRODUCTION

Increasingly, perspectives of people with intellectual disabilities (ID) are included in research concerning their health (Gibbs, Brown, & Muir, 2008; Kuijken, Naaldenberg, Nijhuis‐van der Sanden, & Schrojenstein‐Lantman de Valk, 2016; Young & Chesson, 2006). Regarding health promotion, recent studies provide insights into perspectives of people with intellectual disabilities on enabling and constraining factors for physical activity and healthy nutrition (Cartwright, Reid, Hammersley, & Walley, 2017; Caton et al., 2012; Doherty, Jones, Chauhan, & Gibson, 2018; Kuijken et al., 2016; Spassiani, Meisner, Abou Chacra, Heller, & Hammel, 2019; Temple & Walkley, 2007). These perspectives are helpful in targeting common lifestyle problems among this population such as unhealthy diets, sedentary behaviour and physical inactivity (Adolfsson, Sydner, Fjellström, Lewin, & Andersson, 2008; Hilgenkamp, Reis, van Wijck, & Evenhuis, 2012; Melville et al., 2017). Although people with intellectual disabilities identified the need for a supportive social and physical living environment in these studies, the focus was mainly on individual behaviour and provides little insight into how the setting in which people with intellectual disabilities engage can contribute to healthy living. For people with intellectual disabilities, the setting, for example the social, physical and organizational environment, of intellectual disability support providers plays a key role in health promotion (Marks & Sisirak, 2014; O’Leary, Taggart, & Cousins, 2018).

Existing health promotion for people with intellectual disabilities tends to focus on programme‐based interventions aimed at individual behaviour and not on health promotion in settings where day‐to‐day lifestyle choices are made (Kuijken et al., 2020; Naaldenberg, Kuijken, van Dooren, & de Valk, 2013). These programmes are often short term and therefore fail to become embedded in organizational policy after the programme ends (Kuijken et al., 2020). An exception is the study of Marks and colleagues who attempted to integrate their programme “Health Matters” into daily routines of people with intellectual disabilities and train support staff to support their physical health (Marks, Sisirak, Magallanes, Krok, & Donohue‐Chase, 2019). Although this programme attempts to integrate the activities in daily routines and provide social support for participants, it is not targeted on the setting itself. Only a few studies in health promotion for people with intellectual disabilities have adopted a focus on the setting of intellectual disability support providers. These point out factors that hinder the implementation of health promotion, including a limited health promotion culture, lack of clarity among staff on roles and responsibilities regarding health promotion, and lack of health promotion capacity in intellectual disability support providers (Kuijken et al., 2018; O’Leary et al., 2018; Spassiani et al., 2019). As settings in which people with intellectual disabilities engage play a key role in promoting a healthy lifestyle (Marks & Sisirak, 2014; O’Leary et al., 2018), a broader understanding of how factors in the setting can contribute towards a healthy lifestyle is vital for applying integrated multi‐level health promotion interventions for people with intellectual disabilities and creating sustainable effects (Kuijken et al., 2018; Marks & Sisirak, 2014; Steenbergen, Van der Schans, Van Wijck, De Jong, & Waninge, 2017).

Setting approaches to health promotion is in line with principles from systems thinking where the focus is on understanding the influence of the context and involved stakeholders in how behaviour patterns are created and sustained (Hawe, 2015; Naaldenberg et al., 2009). Rather than focusing on “fixing” one part of the system (being the whole of the issue or problem), the aim is to create a system that allows for healthy behaviour to “emerge” (Fletcher et al., 2016; Hawe, 2015; Rosas, 2015; Rutter et al., 2017). This requires insight in how actors and context relate to each other within the system and highlights the importance of involving all stakeholders (including end‐users) as they have intimate knowledge of the system in everyday practice (Moore & Evans, 2017).

An health promotion approach in which system thinking is adopted is the healthy settings approach, an integrated approach aimed at creating continuous attention on health promotion in the living environment (Rosas, 2015). The approach is underpinned by socio‐ecological theory and organizational change theory (McLeroy, Bibeau, Steckler, & Glanz, 1988; Mittelmark et al., 2017). It was developed in the 1980s and has been a priority of the World Health Organization (WHO) ever since the 1986 Ottawa Charter for Health Promotion (WHO, 1986). It is applied in different settings, for example the Healthy Cities and Healthy Schools programmes (Barnekow Rasmussen & Rivett, 2000; De Leeuw, 2009). This whole‐systems approach aims to understand the relationship between individual behaviour and environmental conditions for health by considering multiple sources of influence. It is focused on embedding health in the routines and culture of a setting (Dooris, 2013). Identifying assets within a setting can enhance the setting’s capacity to promote healthy living (McKnight & Kretzmann, 1993). Assets are protective or promoting factors that maintain and sustain health and wellbeing in a setting, such as skills of individuals, friendship networks, money and schools (Morgan & Ziglio, 2007).

To facilitate intellectual disability care settings to become health‐promoting systems that stimulate healthy behaviour, it is helpful to gain user‐perspectives on structural contributors to physical activity and healthy nutrition in intellectual disability care settings. This study aims to answer the following research question: “What assets for physical activity and healthy nutrition do people with moderate intellectual disabilities and proxy informants of people with severe/profound intellectual disabilities identify and prioritize?”

## 2 METHOD

### 2.1 Context

This study was conducted in the Netherlands and focused on people with moderate to profound intellectual disabilities who receive support from care providers specializing in people with intellectual disabilities. The support for this population includes personal, daily, social and home health tasks, mainly provided by daily care professionals who are paid carers trained in behaviour aspects and/or assistant nursing (Heutmekers et al., 2016). In 2017, about 68,000 people with intellectual disabilities lived in facilities provided by intellectual disability care providers (ZorginstituutNederland, 2019), ranging from clustered group homes to small group living in apartments, and single‐family homes in neighbourhoods (Van, Staalduinen, & ten Voorde, 2011).

### 2.2 Inclusive approach

This study actively involves people with intellectual disabilities as co‐researchers in all stages, following Frankena et al. (2018) guidelines in the consensus statement for inclusive health research. This was used to deploy experiential and scientific knowledge and contribute to appropriate data collection, data quality and relevant outcomes (Frankena et al., 2018; Johnson, Minogue, & Hopklins, 2014). The research team consisted of researchers with intellectual disabilities (co‐researchers) and without intellectual disabilities, all employed by the university. In weekly meetings, the co‐researchers (initials) and (initials) developed the procedure, data collection method and data analysis, and incorporated feedback from other members of the research team and the project’s advisory group including people with intellectual disabilities, caregivers, health professionals and a manager. Data collection and analysis were conducted by (initials of research team).

**FIGURE 1:**
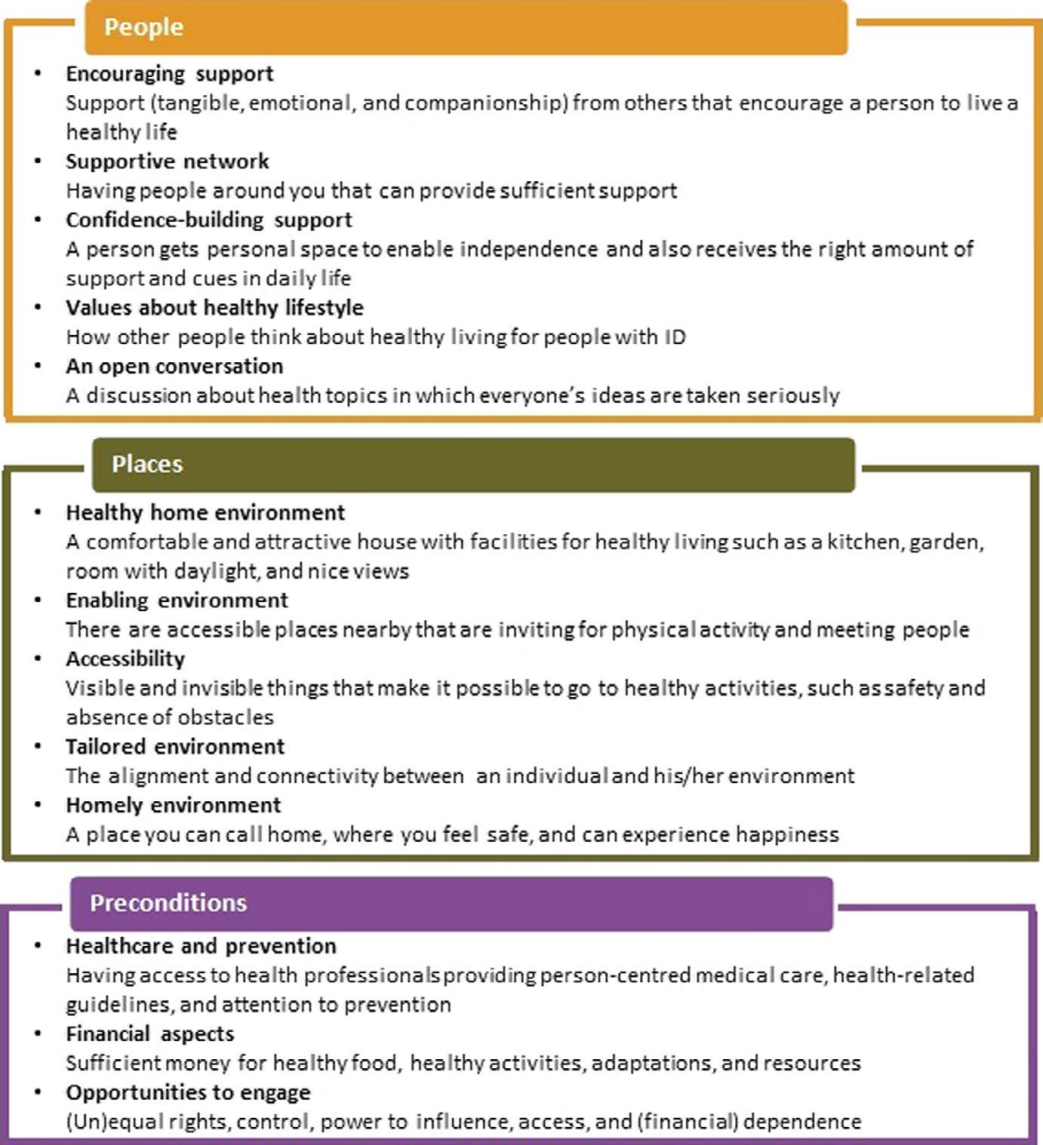
Clusters and overarching themes of the Healthy Settings for People with Intellectual Disabilities (HeSPID) framework

Before the start of this study, co‐researchers expressed the need to better explicate the concept of health‐promoting settings for people with intellectual disabilities and thereby facilitate meaningful data collection. Therefore, a concept mapping study (reference of research team) with researchers specialized in health care for people with intellectual disabilities and researchers specialized in healthy settings was conducted, resulting in the Healthy Settings for People with Intellectual Disabilities (HeSPID) framework described in Figure 1.

Collaboration between the researchers with and without intellectual disabilities was supported by (i) the “research clock,” a clock on which steps of the study were visualized to prompt memory, (ii) a script with points for attention during data collection, (iii) pre‐selected parts of audio‐recordings rather than transcripts for data analysis, (iv) the use of sticky notes during data analysis to visualize generated themes and structure data by placing them on a flipchart based on similarity and (v) verbal explanation of this manuscript to obtain feedback. In addition to this scientific paper, an easy‐read abstract and vlog were written to disseminate the results in an accessible manner.

### 2.3 Procedures

Participants were recruited from 8 intellectual disability care providers. Purposive sampling was used to recruit 4 groups of people with moderate intellectual disabilities and 4 groups of proxy informants of people with severe or profound intellectual disabilities. Adults with moderate intellectual disabilities were able to communicate verbally and lived in accommodation or participated in day activities provided by an intellectual disability care provider. Proxy informants were able to respond on behalf of a person with severe or profound intellectual disabilities whom they had known for at least 6 months and with whom they had weekly contact. Diversity was sought in type of accommodation (living or day activities) and type of proxy (family or care professional). Potential participants received written study information. People with intellectual disabilities were provided with easy‐read information. After stating their interest, written informed consent was obtained. For participants with intellectual disabilities, it was checked whether or not a legal representative should sign the consent form.

The meetings took place between April and August 2018 at a place that was convenient for the participants, mostly in or near their living accommodation. In the meetings with people with moderate intellectual disabilities, the research team consisted of a facilitator (initials), a co‐researcher who assisted in communication (initials) and an observer (initials). In the meetings with proxy informants, the research team consisted of a facilitator (initials) and an observer (initials). If requested by participants with moderate intellectual disabilities, support staff were present.

The study was conducted according to the principles of the Declaration of Helsinki and the EU General Data Protection Regulation. The Medical Research Ethics Committee of Radboud University and Medical Centre approved this study (registration number: 2018–4160).

### 2.4 Data collection

The Nominal Group Technique (NGT) was used to identify and prioritize assets. The NGT is a mixed method to explore expert opinion on a given topic and establish priorities. It has already been used successfully in studies with people with intellectual disabilities (Friedman, Arnold, Owen, & Sandman, 2014; Roeden, Maaskant, & Curfs, 2011; Natasha A Spassiani et al., 2015; Tuffrey‐Wijne, Bernal, Butler, Hollins, & Curfs, 2007). For this study, the NGT was modified to foster meaningful participation of people with intellectual disabilities by splitting the process into two meetings: generating ideas and ranking. After a pilot, small amendments were made to supporting materials.

#### 2.4.1 Generating ideas

Ideas were generated in four rounds. Round one included an open discussion, guided by the question *What in your living environment helps you to be physically active and eat healthily?* Then, three thematic rounds were held on (i) “People,” (ii) “Places” and (iiii) “Preconditions,” relating to the 13 clusters of the HeSPID framework as described in the methods section, see Figure 1 (reference of research team). These thematic round were used to stimulate participants to think about all aspects related to their living environment. At the start of these rounds, pictures relating to the clusters, physical activity and nutrition were explained and visualized. In all rounds, participants were asked to mention all possible assets, for example both existing and desired assets and assets related to themes other than the ones introduced. All participants were stimulated to contribute by giving everyone a turn and using probing questions. The meetings lasted 60–90 min and were audio‐recorded.

#### 2.4.2 Ranking

In the second meetings, participants ranked their group’s ideas in order of importance using a step‐by‐step procedure. Ideas were presented on slips and read out for participants with moderate intellectual disabilities. The participants classified the ideas individually as “important” or “unimportant” by putting the slips in one of two envelopes and compiled a top 5 most important ideas.

### 2.5 Data analysis

Data analysis was conducted through (i) thematic content analysis of audio‐recordings of the idea‐generating meetings with the co‐researchers (initials) using Atlas.ti software 9.2.29 and sticky notes of ideas, and (ii) and statistical analysis of rankings of ideas.

After each idea‐generating meeting, a list of ideas was developed for each group’s ranking meeting, using the following procedure: (i) selecting relevant fragments (initials), (ii) coding relevant fragments and writing down ideas (initials researcher and co‐researchers), (iii) checking analysis (initials) and (iv) finalizing list of ideas (initials of researchers and co‐researchers).

The ideas were thematically analysed independently (*initials of researchers)*. Ideas were grouped and where possible linked to the HeSPID framework (initials of researchers and co‐researchers) (*reference from research group*). Additional categories were allowed to prevent the framework from being restrictive in the analysis. Differences and the “other” category were discussed until consensus was reached. This categorization of ideas by clusters was used for a qualitative description of the gathered ideas, as presented in the results section.

The ranking data were analysed using descriptive statistics. Individual top 5 rankings were transformed into individual scores (e.g., 5 points for first place, 4 points for second place, and so on). The ideas were categorized in clusters to calculate relative importance on cluster level using the formula: (total score for the cluster/maximum points) × 100 (maximum points is calculated as the total number of participants × total points that 1 participant can give) (McMillan et al., 2014). The relative importance on cluster level is presented in the results section for all participants, people with intellectual disabilities and proxy respondents.

## 3 RESULTS

### 3.1 Participants

Table 1 provides an overview of study participants (*n* = 51).

**TABLE 1:** Participant characteristics

	Groups with people with moderate intellectual disabilities	Groups with proxies for people with severe/profound intellectual disabilities
Number of participants (*n*)	21	30
Number of groups (*n*)	4	5
Age of person(s) with intellectual disabilities (range)	21 to 69 years	7 to 83 years
Disabilities of person with intellectual disabilities	Wheelchair bound	Visual impairments
		Hearing impairments
		Physical impairments
		Wheelchair bound
		Behaviour problems
Housing of person with intellectual disabilities	Group home on campus	Group home on campus
	Group home in neighbourhood	Group home in neighbourhood
		With parents
Accommodation for daytime activity for person with intellectual disabilities	Day activity centre, on campus	Day activity in group home
	Day activity centre, in neighbourhood	
	Other (paid jobs)	
Relationship to person with intellectual disabilities	n/a	Parent: 8
		Daily care professional: 9
		Care professional (both daily care and day activity care): 7
		Day activity care professional: 5
		Other (physiotherapist): 1

### 3.2 Generated ideas

The groups generated between 13 and 26 ideas each. The total of 185 ideas overlapped between the groups and fitted mostly within the 13 clusters presented in the framework. One additional cluster was added: *Health‐promoting organizational policies*. The interrelationship between ideas was also discussed by participants.

**FIGURE 2:**
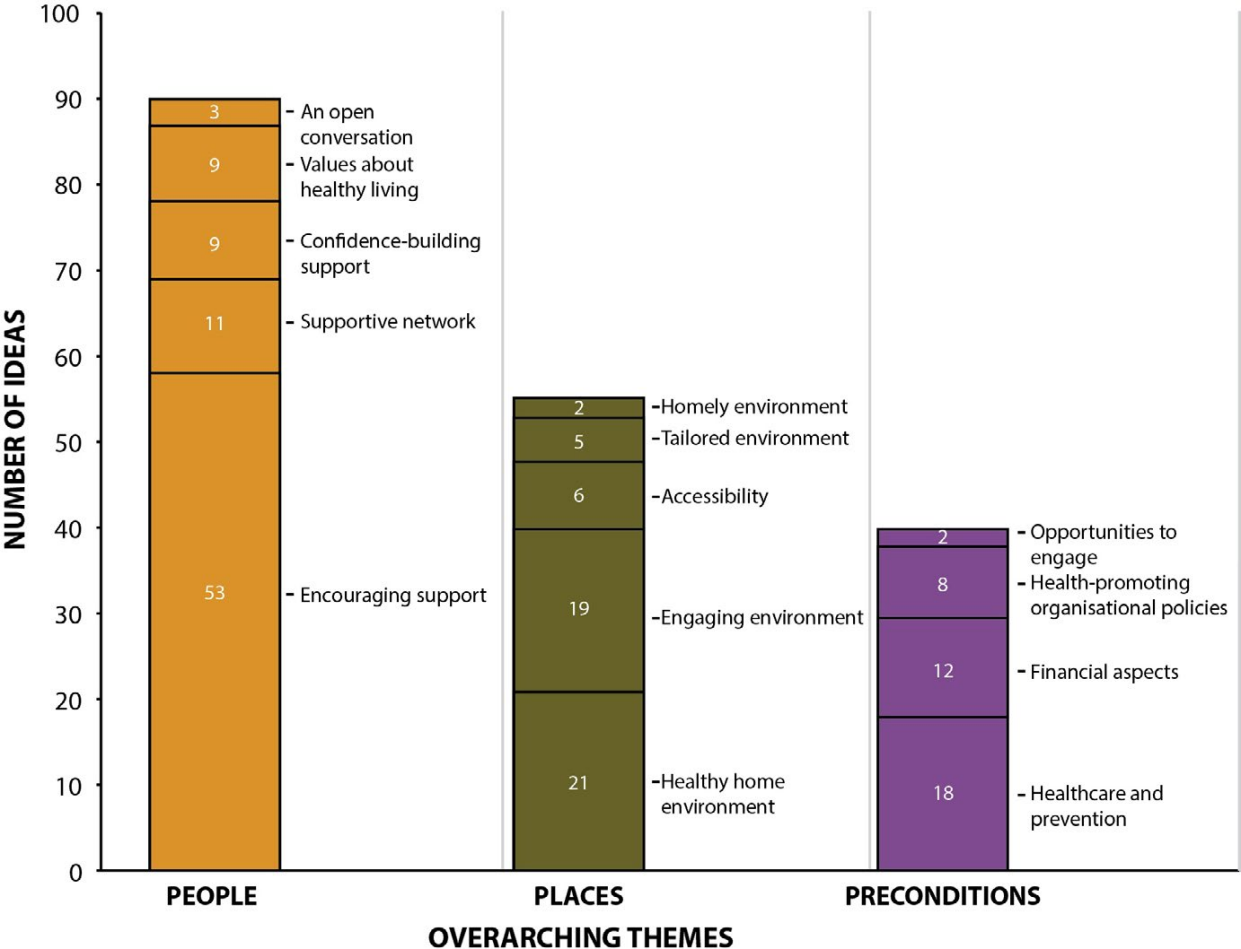
Number of generated ideas per cluster and overarching theme

Figure 2 shows the number of ideas relating to each cluster. About half of the ideas focused on the overarching theme “People” (*n* = 90), with the cluster *Encouraging support* (*n* = 58) including the most ideas. Below, the generated ideas are described for each cluster (*in italics*) and are structured by the overarching themes “People,” “Places” and “Preconditions.”

#### 3.2.1 How “People” can support healthy living

Ideas related to “People” focus on how the social network can support healthy living, the conditions for a stable network and dilemmas in providing support. The participants provided a variety of practical ideas relating to how *Encouraging support* and *An open conversation* can be provided:

1. Emotional support: encouraging healthy nutrition
2. Informational support: providing tips and reminding clients about agreements
3. Tangible support: taking clients to sports facilities, buying healthy foods and providing a balanced diet
4. Providing positive social interactions: cooking healthy meals together, being active in daily life, doing sports together and discussing healthy options
5. Activating clients to be active in daily life: using creative ways to activate clients during the day
6. Showing role model behaviour.

Furthermore, topics mentioned that relate to a *Supportive network* include knowing each other well, continuity of people in the network, enough staff and time to support healthy living. Knowledge, skills, alignment and a shared view of the network regarding healthy living were also mentioned. These factors were often perceived as lacking in the networks of people with intellectual disabilities.

In ideas relating to *Values about healthy living* and *Confidence‐building support,* dilemmas regarding supporting autonomy and healthy living were shared. Different ways of supporting autonomy and balancing this with support for healthy living are illustrated in the following ideas:

1. Making a weekly menu together. For example, care professionals choose the type of meal and clients choose the type of pasta.
2. Clients take turns choosing what they want to eat. Some can choose themselves, and others get help from a care professional who introduces two options. If it is necessary to adjust (because an unhealthy option is chosen), then care professionals do this.
3. Care professionals provide tips for healthy eating and drinking. Clients decide themselves.
4. A balance is sought between quality of life including a client’s preferences and healthy and safe nutrition. For instance, a family can choose to give their child with diabetes more insulin instead of taking away everything he likes and is unhealthy.

#### 3.2.2 How “Places” can support healthy living

How “Places” can contribute to healthy living was reflected in assets relating to tools, facilities, person–environment fit and accessibility. Examples of tools in a *Healthy home environment* include the following: tricycle, interactive tactile wall panel (with movable items to stimulate activity), multi‐sensory stimulation room, hoist, kitchen, vegetable garden and a list with ingredients that clients like/dislike. Other ideas relate to how space in or around a building can stimulate physical activity, for example enough indoor space for physical activities.

In the wider environment, the following facilities were identified as assets for an *Enabling environment*: a swimming pool, supermarket, sports centre, forest, playgrounds and an equestrian centre. Ideas also relate to a beautiful and safe area for physical activity. Demonstrating this, one participant mentioned the idea: “Safe and defined terrain with lots of trees and little traffic where clients can walk freely and do not get lost.”

A good fit between facilities and tools in the physical environment and the needs of people was emphasized as essential. This relates to *Accessibility* and a *Tailored environment*, including suitable activities, flexible opening hours of facilities and accessibility of buildings. For example, one participant with intellectual disabilities mentioned that a cycle path (separated from the road instead of a cycle lane) makes it safer and less scary to cycle to places. Accessibility of the outdoor environment was further reflected in ideas on facilities nearby (such as a supermarket, day‐care, bus stop and park) that can stimulate active forms of transportation, safe routes and accessible forms of transportation. Only two ideas related to *Homely environment*, which focused on feeling safe, accepted and appreciated.

#### 3.2.3 “Preconditions” supporting healthy living

Participants also acknowledged “Preconditions” as assets and gave ideas relating to *Health care and prevention*, *Financial aspects* and *Health‐promoting organizational policies*. Ideas related to *Health care and prevention* include the following: access to medical support and support from allied health professionals by sharing knowledge with care professionals and helping people with intellectual disabilities to live healthily. *Financial aspects* of healthy living as assets focused on several levels: 1) individual budgets for people with intellectual disabilities for physical activity and healthy nutrition, 2) budgets for group homes/day activity centres for healthy nutrition and 3) budgets for care providers to ensure sufficient working hours for care professionals to support healthy living for people with intellectual disabilities and for buying tools for healthy living. Organizational budgets link to ideas on an organization’s policy. Other ideas related to *Health‐promoting organizational policies* include the following: (i) attention on care professionals’ knowledge about healthy living, (ii) discussing healthy living in clients’ personal development plans and (iii) including healthy living in an organization’s vision and mission. Only two ideas related to *Opportunities to engage* and focused on equal treatment and sufficient sports activities tailored to people with intellectual disabilities.

### 3.3 Rankings of ideas

Participants ranked the importance of the ideas individually by compiling a top 5 of the ideas generated in their group. Table 2 shows the relative importance on 14 cluster levels for all participants. The clusters *Encouraging support* (29%) and *Supportive network* (13%) were ranked as most important, followed by ideas related to *Health care and prevention* (9%), *Financial aspects* (8%) and *Healthy home environment* (8%). The cluster *Enabling environment* is remarkable, as it includes many ideas but scores relatively low (6%). The other clusters with a low relative importance (6% or below) include few ideas.

**TABLE 2:** Relative importance of clusters compared by participant type

Cluster	Participants with moderate intellectual disabilities		Proxy informants of people with severe/profound intellectual disabilities		All participants	
	%*	*n* **	%*	*n* **	%*	*n* **
Encouraging support	27	26	30	32	29	58
Health care and prevention	16	6	5	12	9	18
Enabling environment	14	11	1	8	6	19
Healthy home environment	12	7	5	14	8	21
Accessibility	9	7	1	1	4	8
Confidence‐building support	7	5	5	4	6	9
Opportunities to engage	6	2	0	0	2	2
Financial aspects	3	4	12	8	8	12
Tailored environment	3	2	3	3	3	5
Homely environment	3	2	0	0	1	2
Supportive network	1	2	21	9	13	11
Health‐promoting organizational policies	0	0	9	8	6	8
Values about healthy living	0%	0	7%	9	4%	9
An open conversation	0%	3	0%	0	0%	3

* % = Relative importance based on top 5 scores (total score for the cluster/maximum points (participant number × total points that 1 participant can give) × 100).

** *n* = Number of ideas per cluster.

### 3.4 Differences between participants with intellectual disabilities and proxy respondents

Comparison of participants with intellectual disabilities with proxy respondents reveals that there were many commonalities, but also differences in type and relative importance of ideas. Regarding the type of ideas, participants with intellectual disabilities mention practical and visible assets for support, whereas proxy respondents mention more abstract assets and preconditions for support. For example, when looking at *Health care and prevention*, participants with intellectual disabilities mentioned cooking lessons from a dietician and proxy respondents mentioned support from health professionals for care professionals to provide ideas on how to activate people with intellectual disabilities. Also, the ideas of participants with intellectual disabilities related to *Financial aspects* focus on an allowance for groceries, whereas proxy respondents mention attention on healthy living in organizational budgets and policy.

Comparison of the number of ideas per overarching theme reveals that proxy respondents mention more ideas related to “People” (65% vs. 35%) and participants with intellectual disabilities mention more ideas related to “Places” (41% vs. 10%). Both groups mention about the same number of ideas related to “Preconditions” (26% vs. 25%). The relative importance of ideas also differs. The participants with intellectual disabilities ranked *Health care and prevention* (16% vs. 5%) and *Enabling environment* (14% vs. 1%) higher and *Supportive network* (1% vs. 21%) and *Health‐promoting organizational policies* (0% vs. 9%), and *Financial aspects* (3% vs. 12%) lower than the proxy respondents (see Table 2).

## 4 DISCUSSION

This study aimed to identify and prioritize assets for physical activity and healthy nutrition in the living environment of people with intellectual disabilities from their own perspective. The previously developed HeSPID framework supported data collection and analysis (reference from research team). The generated ideas fit well within this framework and highlight the assets that participants deem important for a health‐supporting environment. Most ideas link to the overarching theme “People.” In particular, *Encouraging support,* through activation, role models and regular types of social support, is valued highly. This aligns with the strong dependence of people with intellectual disabilities on others to facilitate healthy living (Kuijken et al., 2018). Care professionals, who are important stakeholders in supporting people with intellectual disabilities to live healthily (Kuijken et al., 2018), lack the prerequisites mentioned as necessary for a *Supportive network*, including knowledge, time and attention on healthy living (Hamzaid, Flood, Prvan, & O’Connor, 2018; Melville et al., 2009; Sundblom, Bergström, & Ellinder, 2015). Ideas generated relating to “Places” provide a clear user perspective on what kind of tools, devices and facilities they consider to be assets that help create a healthy and enabling environment that is accessible and fits their needs (*Tailored environment*). Identified assets related to “Preconditions” elaborated how allied health professionals can contribute to *Health care and prevention* and refined *Financial aspects* into several levels. Furthermore, *Health‐promoting organization policies* were added as a new cluster in the HeSPID framework. Many of the assets mentioned in this cluster, such as organization’s vision and mission, and time and money for assets related to healthy living, are perceived to affect health promotion practice (Robinson, Driedger, Elliott, & Eyles, 2006).

The HeSPID framework distinguishes three overarching themes consisting of 13 clusters. The results from this study indicate that, in practice, identified assets relate to each other within themes and clusters as well as between themes and clusters. For example, to support a person with intellectual disabilities to live healthily (theme “People,” cluster *Encouraging support*), care professionals need knowledge and skills (theme “People,” cluster *Supportive network*), for which an intellectual disability care provider can provide training opportunities (theme “Preconditions,” cluster *Health‐promoting organizational policies*). Participants stressed that this interrelatedness made it difficult for them to rank ideas and consequently difficult to favour one over another. This indicates that, to create a health‐supporting setting for people with intellectual disabilities, an integrated approach is helpful. This is in line with the settings approach to health promotion (Dooris, 2013).

### 4.1 Strengths and limitations

The inclusive approach in which co‐researchers were actively involved is a major strength of this study as this helped to make the right adjustments to the study design for meaningful participation of people with intellectual disabilities as study participants. Lessons learned from the inclusive process include the following: (i) making a protocol with a clear division and instruction of roles and responsibilities of the facilitator and co‐researcher enabled teamwork and helpful support for participants during data collection. Also, analysing the voice recordings to determine ideas and using sticky notes to group ideas helped to work together as co‐researchers and researchers during data analysis. This improved data analysis as experiential, and scientific knowledge was used to interpret the data. However, when considering an inclusive approach, researchers should bear in mind that it takes time and exploration to find ways of working together that contribute to a valuable partnership. The prerequisites and attributes needed for inclusive research, as described in a consensus statement on inclusive research, were helpful in shaping this approach (Frankena et al., 2018).

The adjusted NGT and preparatory study in which the HeSPID framework was developed enabled participants to share their perspective on the abstract term living environment and provided a thorough and diverse overview of assets. The participants stated that the pictures were very helpful. Mentioning the clusters helped them to assess whether a cluster is helpful and to think about ideas (assets) relating to a cluster. Using a pre‐defined framework runs the risk of being too prescriptive and steering the participants. This was mitigated by starting the NGT with an open round before introducing the framework and allowing participants to talk about other themes. The fact that the results altered the original framework by adding a new cluster indicates that this strategy worked well. Although most participants found it easy to value ideas as important or unimportant, many participants found it difficult to compile a top 5 of ideas. This was perceived as difficult by participants with intellectual disabilities because they could choose only 5 out of many important ideas. Proxies also found the task difficult because of the interrelationship between ideas.

To gather perspectives of people with severe and profound intellectual disabilities, the present authors could use only proxy reports. Although this could be seen as a study limitation, as proxy informants cannot truly reflect the voice of people with intellectual disabilities (Scott & Havercamp, 2018), the proxy respondents were able to point out underlying factors that are necessary to create the assets that people with intellectual disabilities mention as needed. The differences in ranking between proxies and participants with intellectual disabilities, however, indicate that using only proxy respondents would have yielded a perspective that was too narrow. This highlights the importance of adjusting research methods to enable people with intellectual disabilities to participate in research.

The context in which support for people with intellectual disabilities takes place is diversely organized across the globe. As this study was executed in the Netherlands, it focuses on the Dutch context in which intellectual disability care providers play an important role in the lives of people with moderate to profound intellectual disabilities. Nevertheless, the HeSPID model was developed in an international context and the results of this study fit well in this model. Applying the HeSPID model and method used in this study in other countries will provide insight in the similarities and differences of assets in other contexts.

### 4.2 Implications for practice

To work towards healthy intellectual disability support settings in practice this study points out implications on governmental, organizational, interpersonal and intrapersonal level. In the last decades, more attention has come for environmental and systems influences on lifestyle, such as how the obesity epidemic is sustained by obesogenic environments (Alvaro et al., 2010). To move beyond an individual focus on health promotion and create system change, governmental policy is critical (Alvaro et al., 2010). When governments want to contribute to healthy intellectual disability care settings, it is pivotal they also gain insight in environmental factors. This study provides key factors to investigate in care settings in order to identify assets and challenges that can be addressed.

To help intellectual disability care providers create a promotion ethos and increase knowledge and time for health promotion, which are currently lacking (Hamzaid et al., 2018; Melville et al., 2009; O’Leary et al., 2018; Sundblom et al., 2015), this study provides points of attention that organizations can use. These include the following: (i) specific attention on care professionals’ professional development, (ii) protected time for health promotion by care professionals, (iii) tools and facilities that are accessible and fit the needs of people with intellectual disabilities and (iv) linking health promotion to personal and organizational values. These factors align with Robinson and colleagues’ points of advice for capacity building (Robinson et al., 2006). More specifically, organizations can use the overview of assets to gain insight in the availability and user‐perspectives of these assets in the context of their organization which serves as input for a health promotion policy and a context‐specific strategic action plan (Marks & Sisirak, 2014).

On inter‐ and intrapersonal level, more attention for health promotion in education for people with intellectual disabilities, their families and care professionals can increase their awareness of the importance of healthy living for health and wellbeing and the different ways in which the environment influences lifestyle choices. They can use this to identify what changes they wish to see in the environment and address these at organizational level. A structured tool based on the study results might be helpful to gather these ideas.

### 4.3 Future research

Future research could identify ways in which people with intellectual disabilities can be involved and empowered in (re)shaping their own living environment. This inclusive study provides an example of how perspectives of people with intellectual disabilities on assets can be gathered, for which the HeSPID model can be a guide. However, tools are needed on how to involve them in the process of (re)shaping their living environment. Furthermore, the identified assets provide context factors which are helpful for development and sustainable embedment of interventions to facilitate healthy behaviour in the system of intellectual disability support settings (Moore & Evans, 2017). Future studies could use these context factors to better understand contextual influences on implementation outcomes and determine what works for whom and under which circumstance (Fletcher et al., 2016; Moore & Evans, 2017; Pfadenhauer et al., 2017).

## 5 CONCLUSION

This study provides a user perspective on assets for physical activity and healthy nutrition in intellectual disability care settings, and thereby also practical implications of the HesPID framework for health promotion practice. The interlinked assets identified can be used in an integrated approach to enhance an intellectual disability care setting’s capacity to promote health and focus on 1) building the capacity of a health‐promoting social network for people with intellectual disabilities, 2) tools and facilities that are accessible and fit the needs of people with intellectual disabilities and 3) capacity building on the organizational level to create a health promotion ethos and (re)orient assets towards health promotion. So, the results provide insight in contextual factors needed for development and sustainable embedment of health promotion in the systems of intellectual disability support settings.

## Ethics approval

The study is conducted according to the principles of the Declaration of Helsinki (October 2013, 64th WMA General Assembly) and in accordance with the EU General Data Protection Regulation. Written informed consent was obtained from all participants prior to data collection. The accredited Medical Research Ethics Committee of the Radboud University and Medical Centre approved the study (registration number: 2018–4160).
